# Receipt of PARP Inhibitors in Patients With Metastatic Prostate Cancer Harboring *BRCA1/2* Alterations

**DOI:** 10.1001/jamanetworkopen.2025.34968

**Published:** 2025-10-02

**Authors:** Micah Ostrowski, Yeonjung Jo, Chadi Hage Chehade, Zeynep Irem Ozay, Georges Gebrael, Nicolas Sayegh, Edwin Lin, Ayana Srivastava, Abigail Gordhamer, Richard Ji, Haoran Li, Vinay Mathew Thomas, Sumati Gupta, Irbaz Bin Riaz, Benjamin L. Maughan, Soumyajit Roy, Neeraj Agarwal, Umang Swami

**Affiliations:** 1 Division of Medical Oncology, Department of Internal Medicine, Huntsman Cancer Institute, University of Utah, Salt Lake City; 2 Cancer Biostatistics, Huntsman Cancer Institute, University of Utah, Salt Lake City; 3 Division of Medical Oncology, Department of Internal Medicine, University of Kansas Cancer Center, Westwood; 4 Division of Hematology and Medical Oncology, Mayo Clinic, Scottsdale, Arizona; 5 Department of Radiation Oncology, UH Seidman Cancer Center, Case Western Reserve University, Cleveland, Ohio

## Abstract

**Importance:**

Patients with metastatic castration–resistant prostate cancer (mCRPC) harboring *BRCA1/2* alterations are eligible to receive poly(adenosine diphosphate-ribose) polymerase (PARP) inhibitors as single agents or in combination with an androgen receptor pathway inhibitor after these agents showed survival improvement in their landmark clinical trials. However, data are limited regarding the uptake of PARP inhibitors in these patients.

**Objective:**

To investigate the use of PARP inhibitors in patients with mCRPC harboring *BRCA1/2* alterations.

**Design, Setting, and Participants:**

This retrospective cohort study used the deidentified Flatiron-Health electronic health record–derived database of US community and academic practices to extract patient-level data. The data cutoff date was May 31, 2024. Patients with mCRPC with evidence of harboring *BRCA1/2* alterations and alive after August 15, 2020 (ie, 3 months after the approval of the first PARP inhibitor, rucaparib, in mCRPC) were included. Statistical analysis was performed from September 2024 to May 2025.

**Exposures:**

Age, race and ethnicity, insurance status, and practice type at the time of mCRPC diagnosis.

**Main Outcomes and Measures:**

The receipt of PARP inhibitors. Multivariable logistic regression was conducted to assess the association between the exposures and the main outcome.

**Results:**

Of 24 105 patients with metastatic prostate cancer, 443 male patients (median [IQR] age, 72 [65-79] years) had mCRPC with *BRCA1/2* alterations and were eligible and included in our analysis. Of these patients, 227 (51.2%) received a PARP inhibitor, whereas 216 (48.8%) did not. Compared with patients covered by a commercial health plan, those covered by Medicare were significantly more likely to receive a PARP inhibitor (odds ratio, 1.91; 95% CI, 1.02-3.66; *P* = .047). The odds of receiving a PARP inhibitor were not significantly higher in patients treated in community practice compared with those treated in academic centers (odds ratio, 1.64; 95% CI, 1.00-2.70; *P* = .05).

**Conclusions and Relevance:**

This retrospective cohort study of patients with mCRPC and evidence of *BRCA1/2* alterations found that approximately half of patients did not receive PARP inhibitors despite evidence of survival improvement in this population. These findings highlight the need to increase awareness of the survival data and access to life-prolonging therapies in patients with mCRPC.

## Key Points

**Question:**

How often are poly(adenosine diphosphate-ribose) polymerase (PARP) inhibitors used in patients with metastatic castration–resistant prostate cancer (mCRPC) harboring *BRCA1/2* alterations?

**Findings:**

In this cohort study of 443 patients with mCRPC and *BRCA1/2* alterations, 51.2% received a PARP inhibitor, whereas 48.8% did not. Patients with Medicare insurance had higher odds of receiving a PARP inhibitor.

**Meaning:**

These findings suggest that despite the availability of biomarker-selected life-prolonging therapies, a sizeable number of patients with mCRPC and *BRCA1/2* alterations do not receive PARP inhibitors, highlighting the need to improve awareness of the data and access to these agents.

## Teaser

This cohort study assesses the use of poly(adenosine diphosphate-ribose) polymerase (PARP) inhibitors in patients with metastatic castration–resistant prostate cancer harboring *BRCA1/2* alterations.

## Introduction

Prostate cancer is a clinically heterogeneous disease, with outcomes varying by specific genomic alterations. The homologous recombination repair (HRR) pathway plays a key role in maintaining genomic stability by accurately repairing double-strand DNA breaks, thereby preventing genetic alterations and chromosomal rearrangements that could potentially lead to carcinogenesis.^1^ Alterations in this pathway are detected in approximately 25% of patients with advanced prostate cancer and are associated with a poor prognosis in the metastatic castration–resistant prostate cancer (mCRPC) setting.^2^ *BRCA1* (OMIM 113705) and *BRCA2* (OMIM 612555) (*BRCA1/2*) alterations are the most prevalent subset of HRR alterations, encountered in 13% of patients with prostate cancer, increase the tumor’s susceptibility to poly(adenosine diphosphate-ribose) polymerase (PARP) inhibitors, and are associated with the highest magnitude of survival advantage with PARP inhibitors.^1,3^

PARP inhibitors demonstrated substantial survival improvement in their respective phase 3 trials and garnered regulatory US Food and Drug Administration approval in biomarker-selected patients with mCRPC either as combinations with an androgen receptor pathway inhibitor (ARPI) or as single agents.^4,5,6,7,8^ Among HRR alterations, alterations in *BRCA* genes are associated with the highest magnitude of survival benefit.^9^ However, data on the uptake of these therapies in patients with mCRPC and *BRCA1/2* alterations outside clinical trials remain limited. In this study, we sought to assess the use of PARP inhibitors in a large US-based dataset.

## Methods

Patient-level data were extracted from the deidentified (US-based) Flatiron Health electronic health record–derived database.^11^ This longitudinal database contains nationally representative data curated via technology-enabled abstraction, primarily from community settings, spanning from 2011 to the present. During the study period, the deidentified data were sourced from approximately 280 cancer clinics, encompassing around 800 sites of care. This retrospective cohort study received approval from the Institutional Review Board at the University of Utah and follows Strengthening the Reporting of Observational Studies in Epidemiology (STROBE) reporting guideline.^10^ Informed consent was not required because the data were deidentified.

The analytic cohort included patients with mCRPC and evidence of alterations in *BRCA1/2* detected on next-generation sequencing testing performed on tumor tissue, blood, or saliva and who were alive after August 15, 2020 (ie, 3 months after the approval of the first PARP inhibitor, rucaparib, in mCRPC), with available treatment information. The data cutoff date was May 31, 2024. Patients were categorized into 2 groups based on the receipt of any PARP inhibitor (ie, olaparib, rucaparib, niraparib, or talazoparib) or not. Baseline characteristics at the time of mCRPC diagnosis, including age; race and ethnicity, which included Asian, Black, Hispanic, White, and other (Alaska Native, American Indian, Native Hawaiian, Other Pacific Islander who are not Hispanic or Latino, or multiracial); insurance plan (commercial health plan, Medicare or other government programs, Medicaid, or others); and practice type (academic or community), were collected in both cohorts.

Race and ethnicity data were obtained from deidentified electronic health records as entered by clinical teams. This information is typically collected from patients during intake interviews and forms, although collection methods may vary across practices. Race and ethnicity data were included in the analysis to assess whether there was an association between race and ethnicity and the receipt of PARP inhibitors.

### Statistical Analysis

A multivariable logistic regression was conducted to compare the odds of receiving a PARP inhibitor in patients with mCRPC harboring *BRCA1/2* alterations. The multivariable analysis included age, race and ethnicity, insurance status, and practice type. The *P* values were calculated using the Wald test, and 95% CIs were calculated using the profile likelihood method. A 2-sided *P* < .05 was used to determine statistical significance, and all the analyses were performed using R, version 4.4.2 (R Foundation for Statistical Computing).^12^ Statistical analysis was performed from September 2024 to May 2025.

## Results

**Figure 1.**
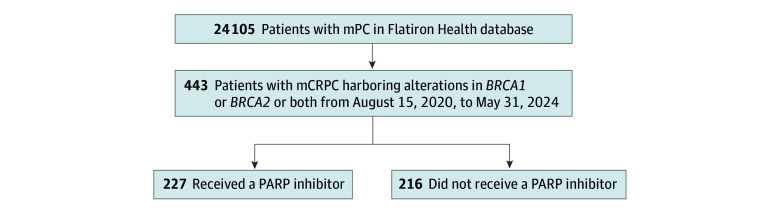
Schematic Representation of the Cohort Selection mCRPC indicates metastatic castration–resistant prostate cancer; mPC, metastatic prostate cancer; PARP, poly(adenosine diphosphate-ribose) polymerase.

Among the 24 105 patients with metastatic prostate cancer in the Flatiron-Health database, 443 male patients (median [IQR] age, 72 [65-79] years; 10 [2.3%] Asian, 52 [11.7%] Black, 20 [4.5%] Hispanic, 273 [61.6%] White, 35 [7.9%] other, and 53 [12.0%] unknown) with mCRPC, evidence of *BRCA1/2* alterations, and who were alive after August 15, 2020, were eligible and included in our analysis. Of these patients, 227 (51.2%) received a PARP inhibitor (166 [73.1%] as monotherapy, 40 [17.6%] in combination with an ARPI, and 21 [9.3%] in combination with other agents) (Figure 1). Among patients who received a PARP inhibitor, 9 (4.0%) were Asian, 22 (9.7%) Black, 10 (4.4%) Hispanic, 149 (65.6%) White, and 37 (16.3%) other or unknown. Among patients who did not receive a PARP inhibitor, 1 (0.5%) were Asian, 30 (13.9%) Black, 10 (4.7%) Hispanic, 124 (57.4%) White, and 51 (23.6%) other or unknown. Other baseline characteristics are given in the Table.

**Table.** Baseline Characteristics of Patients With Metastatic Castration–Resistant Prostate Cancer Who Did and Did Not Receive a PARP Inhibitor

Characteristic	No. (%) of patients	
	Received PARP inhibitor (n = 227)	Did not receive PARP inhibitor (n = 216)
Age, median (IQR), y	72 (65-79)	72 (66-78)
Race and ethnicity		
Asian, non-Hispanic	9 (4.0)	1 (0.46)
Black, non-Hispanic	22 (9.7)	30 (13.9)
Hispanic	10 (4.4)	10 (4.6)
White, non-Hispanic	149 (65.6)	124 (57.4)
Other^a^	13 (5.7)	22 (10.2)
Unknown	24 (10.6)	29 (13.4)
Insurance		
Commercial health plan	192 (84.6)	182 (84.3)
Medicare or other government program	31 (13.7)	18 (8.3)
Medicaid	1 (0.4)	3 (1.4)
Other	2 (0.9)	8 (3.7)
Unknown	1 (0.4)	5 (2.3)
Practice type		
Academic	41 (18.1)	48 (22.2)
Community	186 (81.9)	168 (77.8)
PARP inhibitor regimen		
Monotherapy	166 (73.1)	NA
Combination with ARPI	40 (17.6)	NA
Combination with other agent	21 (9.3)	NA

Abbreviations: ARPI, androgen receptor pathway inhibitor; NA, not applicable; PARP, poly(adenosine diphosphate-ribose) polymerase.

^a^ Alaska Native, American Indian, Native Hawaiian, and Other Pacific Islander who are not Hispanic or Latino or multiracial.

**Figure 2.**
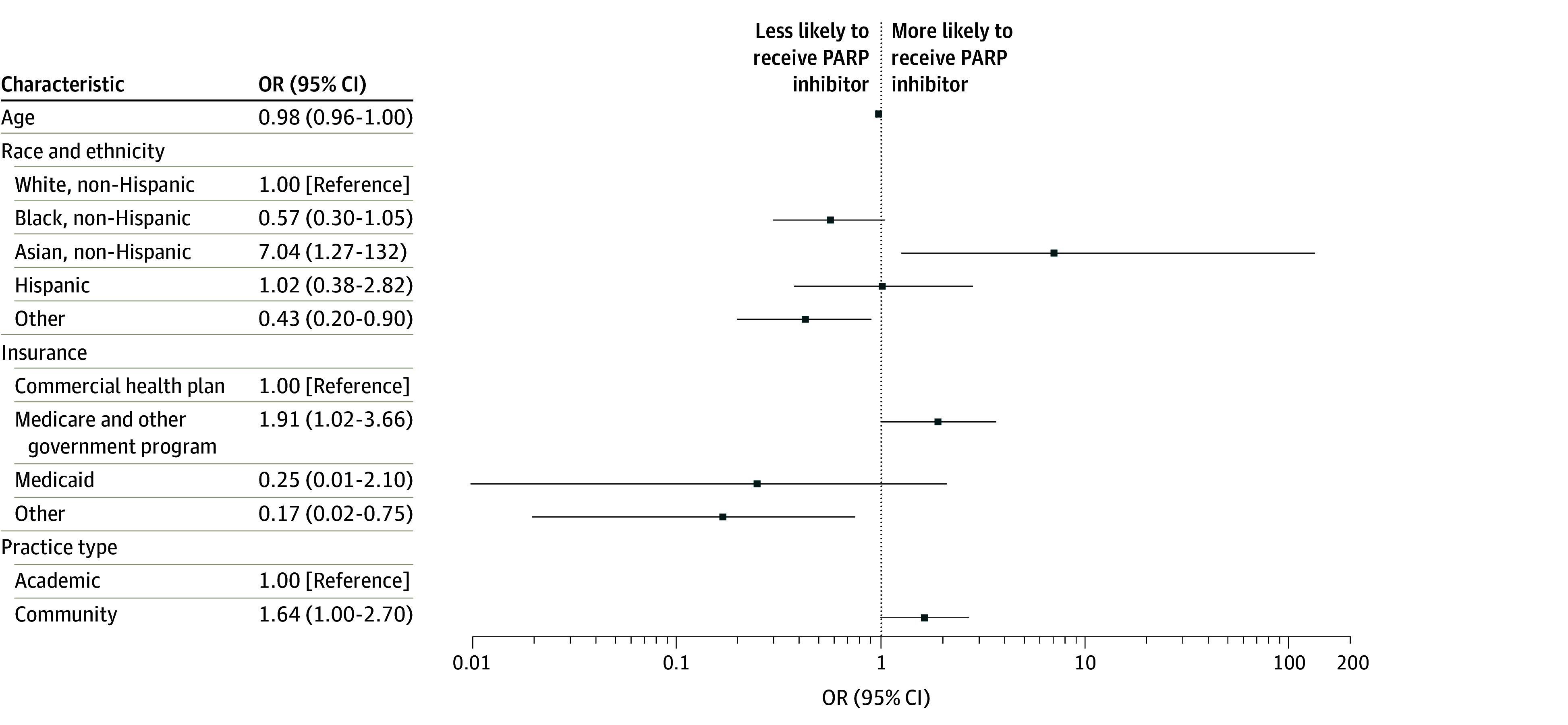
Multivariable Logistic Regression Analysis Showing the Odds of Receiving a Poly(Adenosine Diphosphate-Ribose) Polymerase (PARP) Inhibitor by Different Exposures Other race and ethnicity includes Alaska Native, American Indian, Native Hawaiian, Other Pacific Islander who are not Hispanic or Latino, or multiracial. OR indicates odds ratio.

The results of the multivariable logistic regression model, including age, race and ethnicity, insurance, and practice type, are shown in Figure 2. We did not find any significant association between age and the receipt of PARP inhibitors (odds ratio [OR], 0.98; 95% CI, 0.95-1.00; *P* = .10). Compared with White patients, Black patients were not statistically significantly less likely to receive a PARP inhibitor (OR, 0.57; 95% CI, 0.30-1.05; *P* = .07), and Asian patients (OR, 7.04; 95% CI, 1.27-132; *P* = .07) were not statistically significantly more likely to receive a PARP inhibitor. Compared with patients covered by a commercial health plan, those with Medicare or other government programs were significantly more likely to receive a PARP inhibitor (OR, 1.91; 95% CI, 1.02-3.66; *P* = .047). We also found no significant association between receipt of PARP inhibitors and practice type. Compared with patients treated in an academic practice, those treated in community centers were not statistically significantly more likely to receive a PARP inhibitor (OR, 1.64; 95% CI, 1.00-2.70; *P* = .05).

## Discussion

To our knowledge, this is one of the largest studies assessing the uptake of PARP inhibitors in patients with mCRPC harboring *BRCA1/2* alterations. Our findings suggest that nearly half of these patients are not treated with PARP inhibitors. Additionally, we found that patients covered by Medicare are more likely to receive a PARP inhibitor compared with those with commercial health plans, after controlling for age, race and ethnicity, and practice type.

This underuse of PARP inhibitors in clinical practice is concerning, particularly in the context of the negative prognostic role associated with *BRCA1/2* alterations. In a large dataset of patients with mCRPC, those harboring *BRCA1/2* alterations had inferior overall survival when treated with a taxane or an ARPI in the first-line mCRPC setting compared with those lacking these alterations.^3^ This unfavorable prognosis underscores the importance of optimizing the use of PARP inhibitors, which were associated with an overall survival benefit in patients with *BRCA1/2* alterations.^13,14,15^ PARP inhibitors are currently undergoing investigation in the earlier metastatic hormone-sensitive setting.^19,20,21^ These data suggest that the use of PARP inhibitors may expand in the foreseeable future, affirming the need to optimize their use in patients with *BRCA1/2* alterations to improve outcomes.

Although our findings may suggest the need to improve education among prescribers on the appropriate use of level 1 evidence, lower receipt of PARP inhibitors in academic centers compared with community centers suggests that factors beyond education may contribute to underuse, including but not limited to financial challenges, patient comorbidities, drug-drug interactions, and the emergence of novel therapies.^16^ We found that patients covered by Medicare or other governmental programs were significantly more likely to receive PARP inhibitors compared with those with commercial insurance, reflecting possible disparities in coverage and affordability. For example, patients who have assistance for their copayment through Medicare or other governmental programs may be more likely to receive these drugs compared with those patients who are not covered by these programs and may rely solely on commercial insurance for their coverage.

Hence, in addition to enhancing the knowledge about the data, improving access is critically important to improve the receipt of PARP inhibitors in these patients. Our analysis also indicates that racial and ethnic disparities may exist and limit access to PARP inhibitors. With the caveat of small sample size, Black patients had a nonsignificantly lower likelihood of receiving PARP inhibitors. These findings are concordant with a previous report that showed significantly lower use of biomarker-directed therapies in Black patients with mCRPC compared with their White counterparts.^17^ Similar trends have been observed in ovarian cancer, with non-Hispanic Black patients less likely than non-Hispanic White patients to receive a PARP inhibitor.^18^ These disparities suggest that systemic barriers may impede access to life-prolonging therapies.

### Limitations

The limitations of our study include its retrospective design, undocumented next-generation sequencing testing, missingness in covariates, and unaccounted confounding in the multivariable model. Additionally, patient-specific factors, such as comorbidities, concomitant medications, patient preference, and performance status, which were not captured in this analysis, may have also influenced decisions regarding PARP inhibitor administration.

## Conclusions

Despite PARP inhibitors demonstrating an overall survival benefit in patients with mCRPC harboring *BRCA1/2* alterations, a sizeable number of patients do not receive these agents. These findings highlight the need to increase awareness of survival data and improve access to life-prolonging therapies in patients with mCRPC.

## Notes

Supplement.
Data Sharing Statement
