# Supplementary material for: Receipt of PARP Inhibitors in Patients With Metastatic Prostate Cancer Harboring BRCA1/2 Alterations
Source: JAMA Netw Open. 2025 Oct 2;8(10):e2534968. doi: 10.1001/jamanetworkopen.2025.34968 (PMC12492053; doi:10.1001/jamanetworkopen.2025.34968)
Supplement: Supplement. — Data Sharing Statement [file jamanetwopen-e2534968-s001.pdf]

Data Sharing Statement

Ostrowski. Receipt of PARP Inhibitors in Patients With Metastatic Prostate Cancer Harboring BRCA1/2 Alterations. *JAMA Netw Open*. Published October 02, 2025. doi:10.1001/jamanetworkopen.2025.34968

Data
Data available: No

Additional Information
Explanation for why data not available: The data that support the findings of this study have been originated by Flatiron Health, Inc. Requests for data sharing by license or by permission for the specific purpose of replicating results in this manuscript can be submitted to dataaccess@flatiron.com.
